# Ternary-Emission Molecularly Imprinted Ratiometric Fluorescence Sensor and Kit for the Rapid and Visual Detection of Enrofloxacin

**DOI:** 10.3390/bios15040226

**Published:** 2025-04-02

**Authors:** Siwu Liu, Jingyi Yan, Dani Sun, Siyuan Peng, Jinhua Li, Huaying Fan

**Affiliations:** 1 School of Pharmacy, Key Laboratory of Molecular Pharmacology and Drug Evaluation (Yantai University), Ministry of Education, Collaborative Innovation Center of Advanced Drug Delivery System and Biotech Drugs in Universities of Shandong, Yantai University, Yantai 264005, China; liujiu645@126.com (S.L.); yjy1091897645@163.com (J.Y.); 2 Coastal Zone Ecological Environment Monitoring Technology and Equipment Shandong Engineering Research Center, Shandong Key Laboratory of Coastal Environmental Processes, Laboratory of Coastal Environmental Processes and Ecological Remediation, Yantai Institute of Coastal Zone Research, Chinese Academy of Sciences, Yantai 264003, China; sun19961212@163.com (D.S.); 17658128539@163.com (S.P.)

**Keywords:** enrofloxacin, molecular imprinting, ratiometric fluorescence, post-imprinting mixing, kit

## Abstract

In this study, a RGB based ternary-emission molecularly imprinted ratiometric fluorescence (MI-RFL) sensor was facilely constructed by using a post-imprinting mixing strategy for the sensitive detection of enrofloxacin (ENR). Upon excitation at 365 nm, the MI-RFL sensor exhibited significant emission peaks at 450, 550, and 620 nm. As the ENR concentration increased, the blue fluorescence generated by ENR in the sensing system gradually intensified, while the red and green fluorescence emitted by the quantum dots in the molecularly imprinted polymers (MIPs) was significantly quenched. Sensing conditions were systematically investigated, including the excitation wavelength, mixing ratio of red/green MIPs, the pH of the buffer solution, and the reaction time. Under the optimal conditions, the developed sensor showed a good linear relationship within the range of 0.25–4 ppm along with obvious color change, with a low detection limit of 0.134 ppm. High selectivity was also attained with an imprinting factor up to 11.65. When applied to real samples of seawater and seafood, the sensor showed good recovery rates of 94.3–126.4% and accuracy with a relative standard deviation of less than 3.97%. Furthermore, the sensor-based kit was easily fabricated and, thus, naked-eye detection of ENR was realized onsite. This study can provide a universal approach for the rapid and visual detection of ENR in complicated matrices.

## 1. Introduction

With the development of human food and chemical industries, antibiotics have been widely applied in the control of bacteria and other microorganisms. In particular, enrofloxacin (ENR) is a commonly used antimicrobial agent in agricultural production. Due to its extensive use in the treatment of aquatic products, it is frequently detected in meat products and seafood [1]. ENR and its metabolites can negatively impact both the health of animals and humans, including via the inhibition of the neurotransmitter γ-aminobutyric acid, excitation of the central nervous system, convulsions, ocular issues, reproductive dysfunction, and gastrointestinal disturbances [2]. Furthermore, due to its poor biodegradability, it can accumulate in both biological organisms and the environment, eventually leading to significant marine pollution [3]. Therefore, achieving sensitive detection of ENR levels in marine environments and seafood is critical.

One of the most commonly used methods for detecting ENR is high-performance liquid chromatography (HPLC) coupled with ultraviolet (UV) detection. This method separates the sample components in a high-pressure liquid system and then uses a UV detector to measure ENR at specific wavelengths [4]. However, the HPLC detection method requires specialized laboratory settings and must be carried out by trained professionals with expertise in analytical chemistry. These limitations make it unsuitable for field applications or for use by non-experts, thus hindering its accessibility and practical utility [5,6,7]. Therefore, the development of on-site rapid detection technology is crucial. On-site rapid detection technology refers to the technology of using portable equipment or reagents to quickly complete the analysis [8]. The core goal is to eliminate the complex process of traditional laboratory analysis and realize real-time, portable, and efficient detection. The core principle of on-site rapid detection technology is miniaturizing the instrument and converting and amplifying the signal. It has the advantages of a fast response, being easy to carry out, and being simple/not involving pretreatment. Fluorescence detection is a common method for rapid detection [9]. Fluorescence detection technology is an analytical method based on the fluorescence phenomenon. The fluorescence signals emitted by a substance under specific excitation light are measured to achieve qualitative and quantitative analysis of the samples using a fluorescence spectrometer. In addition to the ability to quantitatively detect the fluorescence intensity, sensors with different fluorescence characteristics can also emit distinct fluorescence colors when illuminated by a portable UV lamp.

Molecularly imprinted ratiometric fluorescence (MI-RFL) is a detection technology combining molecular imprinting technology (MIT) and ratiometric fluorescence detection technology [10]. MIT consists of the synthesis of polymer materials with specific molecular recognition capabilities [11]. These synthetic polymers are known as molecularly imprinted polymers (MIPs) [12]. MIPs can specifically bind with target molecules, akin to the interaction between biological macromolecules (such as antibodies and enzymes). MIPs combine the advantage of sensitivity, stability simplicity and rapidity of RFL detection, and the attained MI-RFL sensors possess outstanding features, such as high selectivity, high sensitivity, convenience, and speediness [13]. Furthermore, they can accurately and visually detect the concentration of the target substances through multiple color changes [14]. As a result, the sensors are attracting increasing interest and have wide applications.

In this study, an MI-RFL sensor was developed to determine the concentration of ENR by emitting different color fluorescence from quantum dots (QDs) and ENR. This sensor incorporated QDs that emitted red and green fluorescence into the nanopolymers. The strategy of post-imprinting mixing (PIM) can make the reaction simpler and adjust the wider color change range of RFL. ENR inherently possesses blue fluorescence and can interact with MIPs by specific recognition and adsorption, resulting in the mixture of three distinct fluorescence colors to enable the quantitative detection of the ENR concentration in samples. Moreover, the fluorescence–color correspondence for different ENR concentrations can be pre-established when exposed to the same excitation wavelength [14,15]. Portable products can be developed based on the MI-RFL sensor, such as the kit herein, and, thus, the public can conveniently use a portable UV flashlight to detect samples by themselves. Systematic condition investigations and analytical performance validation of the RGB-based MI-RFL sensor were carried out, followed by successful applications for seafood and seawater analysis. Furthermore, based on the present study, various MI-RFL sensing methods can be developed for different chemical pollutants, making the sensors and kits highly versatile with great potential market values.

## 2. Materials and Methods

### 2.1. Reagents and Materials

The ENR, glutathione, 3-mercaptopropionic acid, aminopropyltriethoxysilane (APTES), and tetraethyl orthosilicate (TEOS) were purchased from Macklin Biochemical Technology Co., Ltd. (Shanghai, China). The alcohol, methanol, formic acid, ammonium hydroxide, cadmium chloride, tellurium powder, sodium hydroxide, sodium phosphate dibasic, sodium dihydrogen phosphate, and acetonitrile were supplied by Sinopharm Chemical Reagent Co., Ltd. (Shanghai, China). The sodium borohydride was offered by Sigma-Aldrich (Shanghai, China). All chemicals were of at least analytical grade and were used as received. The ENR reference samples of pork and chicken meat powder were supplied by Meizheng Bio-tech (Beijing, China). Ultrapure water of 18.2 MΩ specific resistance (Millipore, Bedford, MA, USA) was used throughout all the experiments.

The fluorescence spectrophotometer (F-7000, HITACHI, Tokyo, Japan) and UV–Vis spectrophotometer (NanoDrop 2000/2000C, Thermo Fisher Scientific, Waltham, MA, USA) and high-performance liquid chromatograph (Agilent 1260, Agilent Technologies Inc, Santa Clara, CA, USA) were used to record fluorescence emission spectra and UV–Vis absorption spectra, respectively. Morphology evaluation was performed using a transmission electron microscope (JEM-2100, JEOL Ltd., Tokyo, Japan) and scanning electron microscope (Hitachi S-4800, Hitachi, Ibaraki, Japan). Fourier-transform infrared (FT-IR) spectra were obtained on an FT-IR spectrometer (Thermo Scientific Nicolet iS50, Thermo Nicolet Corp., Altrincham, UK). The size distribution was analyzed by dynamic light scattering (Mastersizer 2000F, Malvern, Worcestershire, UK).

### 2.2. Preparation of g-MIPs and r-MIPs

The SiO_2_ nanoparticles were prepared using the Stöber method [16]. The nanoparticles were then washed with ethanol and vacuum-dried to form a powder. Then, 50 mL of deionized water and 30 mL of C_2_H_5_OH were added into a three-neck flask with a capacity of 250 mL and stirred evenly, and then 10 mL of NH_3_H_2_O was added. Then, 20 mL C_2_H_5_OH and 5 mL TEOS were mixed evenly and added to the long-neck funnel. The Mixed solution was added to the three-neck flask at the rate of about 1 s per drop. After dropping and stirring for 8 h, the coarse product of SiO_2_ nanoparticles was obtained.

Next, QDs were synthesized using a hydrothermal method. Here, 40 mg NaBH_4_ and 38.3 mg tellurium powder were added to a 2 mL reaction flask and reacted in ethanol aqueous solution (*v*/*v*, 1.5 mL:0.5 mL) at 40 °C for about 4 h to obtain the solution. Then, 68.4 mg CdCl_2_, 0.182 g GSH/75 μL MPA, and 75 mL deionized water were mixed and stirred evenly. The NaOH solution was adjusted to pH 9.0, N_2_ was introduced for 0.5 h, and 1 mL of prepared the solution was added, heated at 100 °C, and refluxed. green quantum dots (g-QDs) were obtained after about 1 h, and red quantum dots (r-QDs) were obtained after about 36 h.

MIPs were synthesized via a one-pot method. The previously synthesized solution was used to prepare g-MIPs (carrying green QDs) and r-MIPs (carrying red QDs). For g-MIPs, 1 mL of SiO_2_ solution (10 mg/mL), 3 mL of g-QDs, 8 mL of H_2_O, 8 mL of 10 ppm ENR solution, and 37 μL of APTES were added to the flask. After stirring for 1 h, 50 μL NH_3_H_2_O and 50 μL TEOS were added drop by drop and then stirred for 12 h without light. Ethanol was used as an eluent to elute the template molecule. In addition, MIPs were washed three times with deionized water to obtain g-MIPs. The r-MIPs were also synthesized through a similar process, in which r-QDs were added. The synthesized MIPs were stored in a refrigerator at 4 °C for the subsequent construction of sensors and the analysis of real samples.

### 2.3. Construction of a Three-Emission MI-RFL Sensor

Here, 200 μL of g-MIPs with 30 μL of r-MIPs and a volume of 1 mL with 0.1 M PB buffer solution (pH 6.5) were mixed. The spiked ENR concentration was 0–4 ppm, and after 12 min of reaction, the fluorescence spectrum was determined under the conditions of an excitation wavelength of 365 nm and a slit width of 10/10 nm, and fluorescence images were obtained under 365 nm ultraviolet lamp.

### 2.4. Analysis of Real Samples

Three kinds of fish samples were pretreated with the reported seafood pretreatment method [17]. Seawater and three types of fish collected from the nearest ocean (the Yellow Sea, 37°49′10″ N, 119°24′46″ E) were used as detection samples. The extraction method involved grinding the samples and adding a 1:1 formic acid–acetonitrile solution. The mixture was shaken and subjected to ultrasonic extraction, then centrifuged, and the supernatant was collected. The method was used to process the three fish species and seawater samples. After analysis by fluorescence spectrometer, no blue emission was detected at the wavelength of 450 nm, indicating that there was no ENR in the sample. The extraction method was then employed for spiking and recovery experiments, followed by the construction of a fitting curve. The reference samples of pork and chicken meat powder were added with Pb buffer to prepare a 1 mL solution with ENR for detecting the accuracy of the MI-RFL sensor and kit.

High-performance liquid chromatography (HPLC) was used to detect the tail fish samples for verifying the reliability of the MI-RFL sensors. The chromatographic conditions were as follows: C18 (5 µm, 4.6 × 250 mm) column, flow rate of 0.6 mL/min, column temperature of 35 °C, injection volume of 10 µL, fluorescence detector with an excitation wavelength at 280 nm, and an emission wavelength at 450 nm. The mobile phase consisted of acetonitrile and 0.02% formic acid aqueous solution (pH = 3.0), and gradient elution was performed as shown in Table S1. This setup was used to verify the reliability of the MI-RFL method. A fixed quantity of MIPs and PB buffer solution was added, and the final volume was adjusted to 1 mL. The ENR concentration in the sample was then calculated using the standard curve to verify its consistency with the spiked ENR concentration. The concentration of ENR in the samples was then calculated based on the fitting curves to verify whether it aligned with the spiked ENR concentration in the samples.

## 3. Results and Discussion

### 3.1. The Construction and Possible Detection Principle of the MI-RFL Sensor

**Figure 1:**
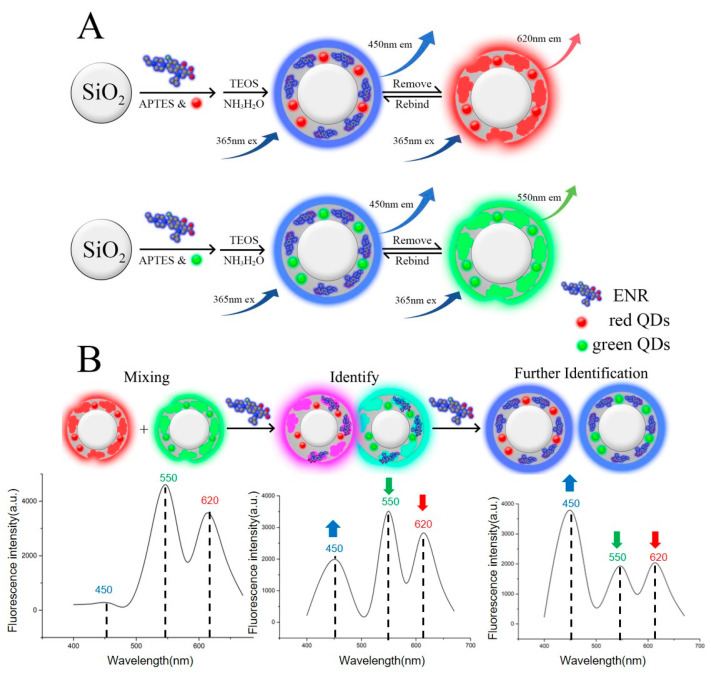
Schematic diagrams of (**A**) the preparation process of r-/g-MIPs and (**B**) the construction and detection procedure of the RGB-based MI-RFL sensor for ENR. In the fluorescence spectra, the dashed lines represent the excitation wavelength at this location, and the arrows represent the changes in fluorescence intensity of this excitation wavelength with increasing ENR concentration.

The MI-RFL sensor with three different color fluorescence was designed and constructed, as schematically shown in Figure 1. With the increase in ENR concentration in the solution, the blue fluorescence of the sensor will continue to increase, while the green and red fluorescence will continue to decrease. The three-emission MI-RFL sensor was constructed using the “imprint first, mix later” strategy [15]. The g-MIPs and r-MIPs were mixed in controlled proportions based on the fluorescence intensity. The red fluorescence with the same intensity as the green fluorescence detected by the fluorescence spectrometer is more prominent, leading to an apparent red appearance and narrowing of the color change range during the visual observation. Therefore, a greater quantity of g-MIPs should be added compared to r-MIPs during the mixing process of the two MIPs. The fluorescence intensity of the system was measured using a fluorescence spectrometer (with an excitation wavelength of 365 nm, a starting wavelength of 390 nm, an ending wavelength of 650 nm, and a slit width 10/10 nm). Finally, ENR was visually detected using a portable ultraviolet light. As shown in Figure 1, the emission fluorescence of the MI-RFL sensor changed with the increase in the ENR concentration in the solution.

As seen from Figure 1, APTES was used as the functional monomer. APTES can couple with SiO_2_ nanoparticles to enhance their adhesion, chemical properties, water resistance, and stability characteristics. TEOS and ammonia were used as cross-linking agents, both of which react well with ENR, fixing it onto the surface of SiO_2_ nanoparticles. The addition speed of the two reagents must be strictly controlled under stirring to ensure uniform attachment of ENR to the SiO_2_ nanoparticle surface. The ENR is then removed from the polymer. According to the reported studies, fat-soluble solvents are more effective for washing out ENR [18]. Ethanol was chosen as an eluent in our experiment. After three elutions, the MIPs solution showed almost no blue fluorescence under the fluorescence spectrometer, indicating that the ENR in the MIPs had been completely eluted. The MIPs were then washed three times with pure water to remove impurities. The resultant cavities left in the MIPs are what we refer to as “imprinted cavities”. These imprinted cavities are highly similar in shape to ENR and contain functional groups that can bind with ENR or similar molecules. In the experiment, non-imprinted polymers (NIPs) were designed as a control. The NIPs were synthesized without adding the ENR solution, using pure water instead. Therefore, the polymer does not have specific imprinted cavities that can recognize ENR, and its detection results were significantly lower than those of MIPs.

Under illumination with a 365 nm ultraviolet light, ENR exhibits an apparent blue fluorescence. When analyzed using a fluorescence spectrometer, it was found that the maximum emission wavelength of this fluorescence is located around 450 nm. The QDs incorporated into the MIPs can be synthesized with varying conditions to adjust their maximum emission wavelength in the fluorescence spectrum. The maximum emission wavelengths of the g-MIPs and r-MIP synthesized in this study were 550 nm and 620 nm, respectively. In this way, the interference of the frequency-doubling peak of the fluorescence spectrometer could be avoided. The blue, green, and red fluorescence emissions could be effectively separated without mutual interference.

The quenching mechanisms of fluorescence are generally classified into two types, namely electron transfer and energy transfer. The reduction in green and red fluorescence may be related to fluorescence resonance energy transfer (FRET) or photoinduced electron transfer (PET) [19]. Data obtained from the fluorescence spectrometer revealed that the three colors of emitted fluorescence exhibited minimal overlap, suggesting that there is no energy overlap between them (Figure S1). According to the literature [20,21], it can be deduced that the charge transfer between QDs and ENR results in fluorescence quenching of the QDs. Electrostatic complexation could occur between the carboxyl moiety of ENR and primary amine moieties on the MIPs surface, facilitated by proton transfer. When ENR is rebound, the charge transfer between the QDs and ENR could cause fluorescence quenching [22]. The reduction in fluorescence intensity of g-QDs/r-QDs was not due to a FRET process but rather due to the PET mechanism from the conduction band of QDs to the lowest unoccupied molecular orbital of ENR, resulting in non-radiative recombination pathways that effectively quench fluorescence emission.

### 3.2. Characterization of SiO_2_, MIPs, and NIPs

**Figure 2:**
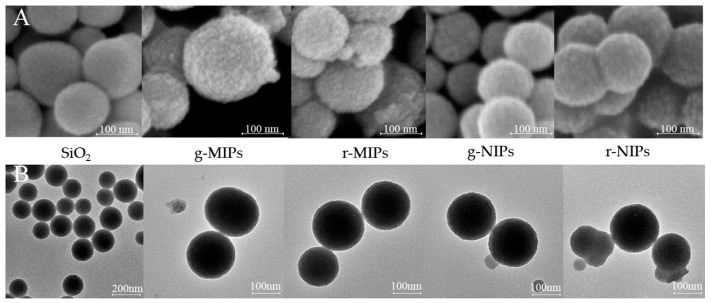
(**A**) SEM images of SiO_2_ and MIPs/NIPs; (**B**) TEM images of SiO_2_ and MIPs/NIPs.

The SEM and TEM were used to observe the morphology and particle size of SiO_2_, g-MIPs, g-NIPs, r-MIPs, and r-NIPs. The MIPs prepared by surface imprinting technology have binding sites for ENR on their surface. Therefore, the surface shell formed must be uniform and moderate in thickness to facilitate ENR binding and provide sufficient binding sites. When the shell is too thick, the increasing mass transfer resistance will result in too slow binding. On the other hand, a too thin shell may lead to template leakage and insufficient binding sites. As shown in Figure 2, the SiO_2_ nanoparticles have an average particle size of approximately 150 nm, with a spherical structure and excellent dispersion. The particle size of the MIPs and NIPs ranges from 170 to 180 nm. They both have an appropraite thin shell of 20–30 nm. The MIPs have a rougher surface compared to NIPs due to the formation of recognition cavities for ENR.

Energy dispersive X-ray spectroscopy (EDS) was also performed on the samples, as shown in Figure S2. It can be observed that the MIPs contain significant amounts of Si, Te, and Cd elements, confirming the successful incorporation of QDs onto the surface of SiO_2_ particles.

The FT-IR analysis was conducted to identify the functional groups in the polymer and determine its composition further. As shown in Figure S3, the characteristic peaks at 510 cm^−1^ and 800 cm^−1^ were attributed to the symmetric stretching vibrations of Si–O. The characteristic peak of CdTe QDs was also at 800 cm^−1^ [23], so the characteristic peak at 800 cm^−1^ of MIPs and NIPs was more significant than that of SiO_2_. The peak at 980 cm^−1^ may be caused by the asymmetric stretching vibration of Si–O–Si, indicating the presence of MIPs and NIPs in the silica-based material. The peak at 1400 cm^−1^ corresponds to the C–O stretching vibration, indicating the presence of COO^−^ groups. The peaks at 3340 cm^−1^ are attributed to the stretching vibrations of –OH and –NH groups. The peaks at 1400 cm^−1^ and 3340 cm^−1^ further confirm the presence of CdTe QDs.

### 3.3. Condition Optimization of the MI-RFL Sensor

Several main factors affecting the MI-RFL sensor were studied, including buffer pH, excitation wavelength, reaction time, and the mixing ratio of r-MIPs and g-MIPs.

#### 3.3.1. The pH of the Buffer

The PB buffer was used to stabilize the pH of the sample at 6.5. Since the solubility of ENR and fluorescence intensity of QDs are influenced by the pH environment, a certain amount of buffer solution was added to stabilize the MIPs and establish a reliable standard curve. Referring to a previous study [24], PB was selected as the optimal buffer system for the MI-RFL sensor based on systematic pH optimization studies. These investigations revealed marked signal attenuation in both strongly acidic and alkaline regimes due to compromised protonation–deprotonation equilibria critical for recognition site functionality [24], resulting in the sensor performing poorly. When the pH was below 5, the fluorescence intensity of the QDs was severely quenched. On the other hand, when the pH was too high, the molecular imprinting shell might have dissociated, leading to surface defects, which ultimately affected the interaction between ENR and the sensor [25]. Therefore, experiments in this study were conducted with a pH gradient of 0.5, covering pH values from 5 to 9, in a total of nine groups. The ratio fluorescence signals of ENR in buffer solutions of different pH values are shown in Figure S4. Based on the results, the ideal fluorescence intensity ratio was obtained at pH 6.5, where both the ENR and quantum dot fluorescence signals exhibited optimal intensity.

#### 3.3.2. Excitation Wavelength

Different excitation wavelengths can significantly affect the fluorescence signal of MIPs. The optimal excitation wavelength for ENR is around 275 nm [26]. However, as shown in Figure S5, the emission wavelengths of green and red fluorescence emitted by MIPs are about 550 and 620 nm, respectively. When the excitation wavelength was 315 nm or lower, the generated frequency doubling peak interfered with the fluorescence emitted by MIPs. In addition, the blue fluorescence emitted by ENR was too strong to produce a good ratio effect with the green and red fluorescence. Considering that the excitation wavelength of most portable UV lamps on the market is 365 nm, and that the increase in excitation wavelength led to the blue fluorescence quenching of the ENR emission, the excitation wavelength was selected at 365 nm.

#### 3.3.3. Reaction Time

The twelfth minute after the sensor was mixed with the sample was determined as the detection time. To prevent time-dependent degradation of the sensor system, the MIPs stored under refrigeration were retrieved for sensor assembly only when required, followed by the immediate introduction of ENR for detection upon sensor construction. A longer reaction time may reduce experimental efficiency and cause the emitted fluorescence to naturally quench. Therefore, an appropriate reaction time must be selected as the standard. Upon adding ENR-containing samples to MI-RFL sensors, a significant decrease in red and green fluorescence intensities is observed. As the reaction time increases, the fluorescence intensities continue to decline and gradually stabilize. As shown in Figure S6, the red and green fluorescence intensities level off, indicating that, at this point, the ENR has fully reacted with MI-RFL sensors, i.e., after approximately 12 min.

#### 3.3.4. Mixing Ratio of g- and r-MIPs

The volume ratio of g-MIPs to r-MIPs was adjusted to 20:3, which made the sensor show the most significant color change under UV light in the linear concentration range of ENR. The pre-experiment results show that more g-MIPs need to be added in the mixing process because the red fluorescence is more prominent than the green fluorescence. The sensor predominantly exhibited a green-dominant optical output when detecting low-concentration ENR analytes. The higher the concentration of g-MIPs, the stronger the quenching effect of ENR on green fluorescence, and the quenching effect of green fluorescence is more obvious than that of red fluorescence. With the increase in ENR concentration, the color of the sensor changes from green to a mixture of red and green/blue intermediate colors, and finally to the blue fluorescence dominated by ENR. As shown in Figure S7, the ratio of g-MIPs to r-MIPs is 20:3, which produces the richest color change effect, as described above.

### 3.4. Sensitivity and Selectivity of the MI-RFL Sensor

**Figure 3:**
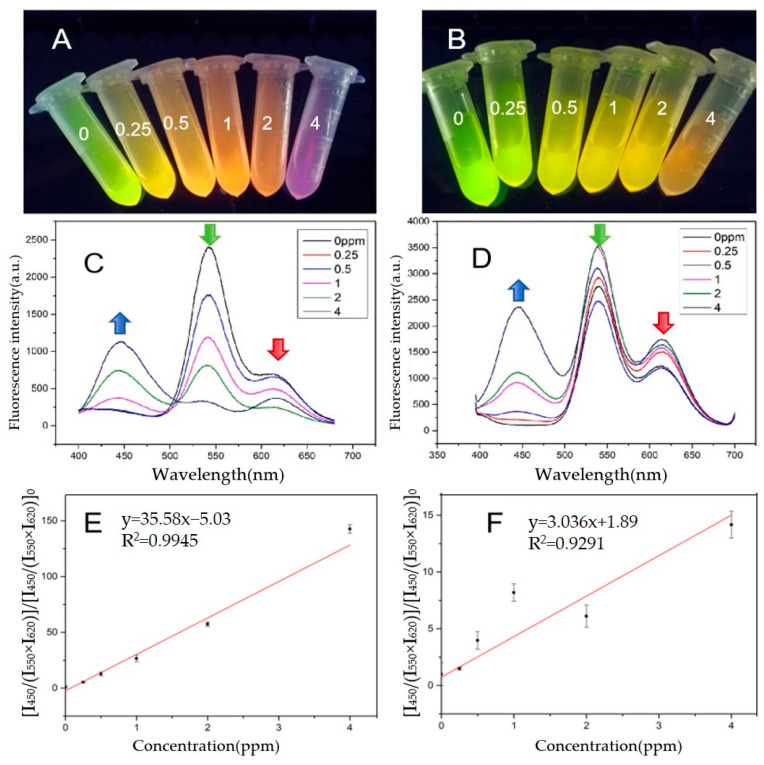
Fluorescent photos of the solutions with ENR from 0–4 ppm detected by (**A**) MI-RFL and (**B**) NI-RFL sensors; fluorescence spectra of (**C**) MI-RFL and (**D**) NI-RFL sensors; corresponding fitted curves of (**E**) MI-RFL and (**F**) NI-RFL sensors. The arrows represent the changes in fluorescence intensity of this excitation wavelength with increasing ENR concentration.

Under optimal conditions, a multi-channel visual detection system based on ternary emission sensors was constructed. As shown in Figure 3A,C, when the ENR concentration was 0, only two fluorescence sources existed at a single excitation wavelength of 365 nm. As the ENR concentration increased, both green and red fluorescence were quenched simultaneously, and the green fluorescence was more significantly affected (Figure 3C). The sensor changed color from green to yellow, then pink, and finally to purple (Figure 3A). When the ENR concentration reached 5 ppm, the red and green fluorescence were almost completely quenched by ENR. The sensor ultimately appeared blue. The ratio of the intensity of the red, green, and blue emission peaks [I_450_/(I_550_ × I_620_)]/[(I_450_/(I_550_ × I_620_)]_0_ showed a good linear relationship with the ENR concentration in the range from 0.25 to 4 ppm, and the correlation coefficient was 0.9945, as shown in Figure 3E. The limit of detection (LOD) was 0.134 ppm, suggesting that the MI-RFL sensor had high sensitivity.

As a control, a three-emission sensor composed of NIPs was constructed. This sensor followed the same conditions, except for the omission of the imprinted target molecule ENR during the synthesis process. Comparing Figure 3C and 3D, the NIPs, due to the lack of an ENR-imprinted cavity, can only partially nonspecifically adsorb ENR in the solution. Consequently, ENR cannot tightly bind to the NIPs, reducing the interaction between the blue, green, and red fluorescence. The intensity ratio of the three fluorescence colors of the NIPs measured showed a lower R-squared value and slope compared with that of the MIPs. The imprinting factor (IF) refers to the ratio of the slope of the calibration curve of MIPs sensor to that of NIPs sensor, which is a critical indicator to measure the quenching effect of MIPs. The IF was as high as 11.65, which indicated the MI-RFL sensor had quite a high specific recognition ability and a high adsorption capacity owing to the presence of a large number of imprinting cavities complementary to the template molecule ENR in size, shape, and functionality. The relative standard deviations (RSDs) of the MI-RFL sensor were 2.40–6.62%, whereas the RSDs of the NI-RFL sensor were 6.05–15.69%, which fully demonstrate that the MIPs sensor had higher precisions and accuracy. The color change in the NI-RFL sensor was not apparent as the ENR concentration increased, as displayed in Figure 3B, from green to light green, olive, pale yellow, yellow, and orange. This was because the NI-RFL sensor lacks specific recognition sites. In contrast, the MI-RFL sensor exhibited profuse color changes when the ENR concentration increased, from green to light yellow, yellow, orange, reddish orange, and purple. Consequently, in comparison, the MI-RFL sensor has a stronger visual detectability. Therefore, the MI-RFL sensor has the remarkable advantages of high sensitivity and precision and strong visual detectability.

**Figure 4:**
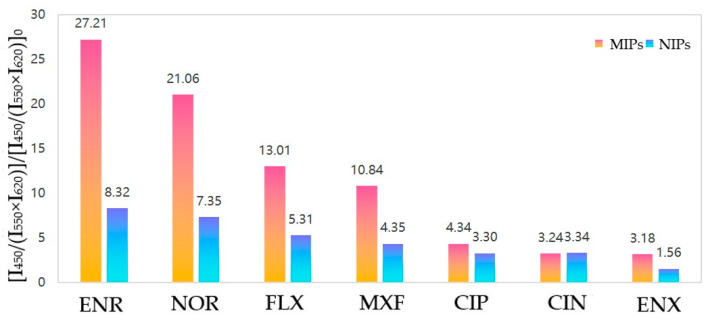
Schematic representation of selectivity testing for different quinolones at 1 ppm concentrations.

The MI-RFL sensor still had the best selection for the target ENR even in FQs with similar structures. As shown in Figure S8, the structures of ENR and other structurally similar FQs that may coexist in actual samples were compared. After adding various structural analogs at the same concentration as ENR, the fluorescence spectra of the sensor were measured. The fluorescence quenching rates of ENR and these analogs were calculated to evaluate the sensor’s selectivity. As shown in Figure 4, ENR exhibited the best quenching effect on MIPs. Some other FQs, such as NOR, also showed notable quenching effects. This could be attributed to the fact that MIPs still have some nonspecific recognition ability for these analogs, as these molecules share similar functional groups and molecular sizes with ENR. These analogs allowed for some level of specific binding [27]. However, the quenching effect of ENR still showed a difference compared to most other FQs. This indicated that the sensor could exhibit greater differentiation when recognizing other types of drugs or chemical molecules. This further demonstrated the strong selectivity and anti-interference capability of the MI-RFL sensor.

### 3.5. Intra- and Inter-Day Variation Study of the MI-RFL Sensor

The intra-day and inter-day experiments of the MI-RFL sensor were conducted to examine the stability of the sensor and thereby the robustness of the sensing method. The constructed MI-RFL sensors were divided into two groups for intra-day and inter-day precision experiments, respectively. On one day, the MI-RFL sensor was used to measure the ENR at three concentration levels of 0.25, 1 and 4 ppm, respectively, with each test repeated. Similarly, over three consecutive days, the MI-RFL sensor was used to measure the ENR at the above three concentrations, separately, five times (each time with an interval of 12 h). As listed in Table S2, the RSDs for the intra-day precisions were between 1.49–2.21%, and the inter-day precisions were 4.14–7.81%, respectively, indicating that the sensor was highly precise. Consequently, the robustness of the sensing method was demonstrated.

### 3.6. Application of the MI-RFL Sensor to Real Samples

To evaluate the performance of MI-RFL sensors in practical applications, marine products and seawater samples from coastal areas near cities were collected. As mentioned earlier, no ENR was detected in the seawater and fish samples during the actual detection process, prompting us to conduct spiking experiments. Three spiking concentrations were established at 0.25, 1, and 4 ppm. As shown in Table 1, the results indicated that the relative standard recovery rate ranged from 94.3% to 126.4%, with RSDs ≤ 3.97%. The MI-RFL sensor had good recovery and repeatability.

To further validate the reliability of this method, the analytical results were compared with that of the HPLC method. Within the linear range of the fitting curves, the tail fish and pomfret fish samples were detected by HPLC-UV at the same time. As shown in Table 2, the average measurement error of tail fish ranged from 6.81% to 7.15% and the average measurement error of pomfret fish ranged from 2.88–5.02%. The reference samples of pork and chicken meat powder samples were detected with measurement errors of 6.30–11.84%, and RSDs of 3.37–0.88%, respectively. These results could fully validate that our developed sensor was quite reliable and practical. Meanwhile, corresponding colorimetric results were shown in Figure S9. For the two fish samples, the fluorescence color changed from green, to light green, yellow, and purple with concentrations from 0 to 4 ppm. The tested pork and chicken sample solutions a exhibited green color. The fluorescence colors of the different real samples were almost consistent with those of corresponding standard solutions, indicating that the visualization ability of MI-RFL sensor could be utilized in real sample analysis. Consequently, the developed MI-RFL sensing method was highly accurate, reliable, and applicable for the quantitation and visual detection of trace ENR in complicated samples.

### 3.7. Method Performance Comparison of MI-RFL Sensor

Method performance of the validated MI-RFL sensor for ENR was compared with that of reported methods [28,29,30,31], as shown in Table S2. As seen, the MI-RFL sensor offers lower LOD and higher precision values than the electrochemistry [30] and HPLC-UV [31] approaches, and more sample analysis and higher visualization effects than previously reported [28,29,30,31]. The higher sensitive methods [28,29] cannot perform visual detection, needing the help of equipment to perform the accurate detection of samples. As such, our MI-RFL sensor possesses the characteristics of high sensitivity, precision, practical applicability, and visualization capability.

### 3.8. Analysis of Real Sample by the MI-RFL Sensor Based Kit

**Figure 5:**
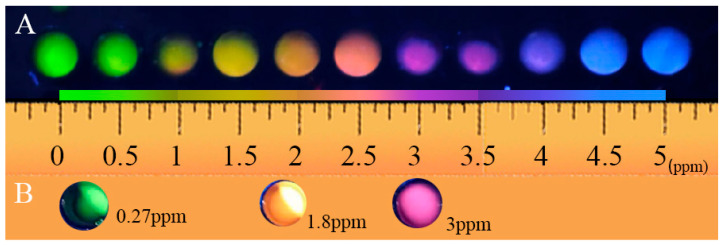
(**A**) Color card fitted with a standard ENR solution; (**B**) color of 0.27 ppm ENR in the pork sample, 1.8 ppm ENR in the chicken sample, and 3 ppm ENR in the tail fish sample, irradiated by a UV flashlight.

The well-developed MI-RFL sensor was then used for constructing a kit for the on-site detection of ENR in real samples. As shown in Figure S10, a kit was attained, with a length of 17 cm, a width of 10 cm, and a height of 10 cm, with well-prepared reagents and materials contained within it. As such, the kit could complete the screening and detection of ENR. The main materials, namely MIPs, were stored in a dry-powder form. Therefore, the reagent kit exhibited excellent storage stability under low-temperature conditions. The concentrations of ENR in different actual samples were detected by the naked eye, including pork samples containing 0.27 ppm ENR, chicken samples containing 1.8 ppm ENR, and tail fish samples containing 3 ppm ENR. The chicken and pork were powder solutions. The fish samples were simply crushed and soaked in ENR solution for 24 h. As shown in Figure 5, the colors of certain concentrations of ENR from actual samples were almost the same as those of the corresponding concentration from the color card. Therefore, the MI-RFL sensor based kit was demonstrated to be feasible for the sensitive, accurate, rapid, and visual on-site detection of trace ENR in complex samples.

## 4. Conclusions

In summary, the MI-RFL sensor and related kit were easily constructed for specifically recognizing and sensitively detecting ENR. This sensor was developed based on the post-imprinting mixing strategy and could emit RGB ternary-emission fluorescence at different ratios according to the different ENR concentrations. The synthesis conditions of this sensor were optimized to improve sensitivity and provide a wider range of color variations through naked-eye observation during the validation experiments using seawater, fish, pork, and chicken as real sample models, indicating that this sensor can accurately detect ENR without the help of laboratory instruments. The kit used for the on-site detection of ENR was manufactured and validated for real sample detection, and the detection results were in line with expectations. The MI-RFL sensor and kit can be expected to play a considerable role in the on-site detection of various analytes in complicated matrices.

## Figures and Tables

**Table 1:** Endogenous ENR content and spike recovery in seawater and seafood samples detected by the MI-RFL sensor (*n* = 3).

Sample	Spiked Concentration (ppm)	Measured Value (ppm)	Recovery (%)	RSD (%)
Seawater	0	ND ^a^	–	–
	0.25	0.290	116.0	0.91
	1	1.171	117.1	0.47
	4	3.857	96.4	1.22
Bream fish	0	ND ^a^	–	–
	0.25	0.311	126.4	0.31
	1	1.089	108.9	0.44
	4	3.773	94.3	0.71
Tail fish	0	ND ^a^	–	–
	0.25	0.282	112.8	2.17
	1	1.021	102.1	3.80
	4	4.244	106.1	2.78
Pomfret fish	0	ND ^a^	–	–
	0.25	0.308	123.5	0.15
	1	1.213	121.3	3.97
	4	4.330	108.3	0.74

^a^ Not detected.

**Table 2:** The comparison of the detection of ENR in tail fish and pomfret fish samples between the MI-RFL sensor and HPLC, and the detection results comparison of ENR in pork and chicken samples between the MI-RFL sensor and the tagged values (*n* = 3).

Sample	Tagged Value (ppm)	Spiked Concentration (ppm)	MI-RFL Sensor (ppm)	HPLC-UV (ppm)	Measurement Error (%)	RSD (%)
Tail fish	–	0	ND ^a^	ND ^a^	–	–
		0.25	0.282	0.303	6.93	2.18 ^b^
		1	1.081	1.160	6.81	1.19
		4	4.244	4.571	7.15	1.05
Pomfret fish	–	0	ND ^a^	ND ^a^	–	–
		0.25	0.308	0.324	4.93	1.88
		1	1.213	1.179	2.88	0.51
		4	4.330	4.559	5.02	0.23
Pork	0.270	–	0.253	–	6.30	3.37 ^c^
Chicken	0.456	–	0.510	–	11.84	0.88

^a^ Not detected. ^b^ The value for HPLC-UV. ^c^ The value for MI-RFL sensor.

## Data Availability

Detailed data can be obtained from the authors.

## Supplementary Materials

The following supporting information can be downloaded at: https://www.mdpi.com/article/10.3390/bios15040226/s1. Figure S1. Fluorescence spectra of ENR, g-MIPs, and r-MIPs excited by 365 nm. Figure S2. Energy-dispersive X-ray spectroscopy analysis: (A) SiO_2_; (B) g-MIPs; (C) r-MIPs; Figure S3. Infrared spectroscopic analysis: (A) SiO_2_; (B) g-MIPs; (C) r-MIPs; (D) g-NIPs and (E) r-NIPs; Figure S4. Effects of pH on ratio fluorescence of MIPs with 1ppm ENR; Figure S5. Fluorescence spectra of MIPs with 1 ppm ENR at different excitation wavelengths; Figure S6. The MI-RFL with time after adding 4 ppm ENR; Figure S7. Effects of mixing volume ratio of green and red QDs on the fluorescence color of MIPs; Figure S8. The structural formula of ENR and its analogs; Figure S9. Fluorescence color results of different samples containing different concentration ENR; Figure S10. Photo of kit packaging and contents; Table S1. Mobile phase elution method; Table S2. Intra-day and inter-day precisions of MI-RFL sensor; Table S3. Comparison of the analytical performance of different MIPs based methods for ENR detection.
