# Supplementary material for: Ternary-Emission Molecularly Imprinted Ratiometric Fluorescence Sensor and Kit for the Rapid and Visual Detection of Enrofloxacin
Source: Biosensors (Basel). 2025 Apr 2;15(4):226. doi: 10.3390/bios15040226 (PMC12024939; doi:10.3390/bios15040226)
Supplement: Supplementary file 1 [file biosensors-15-00226-s001.zip › biosensors-3520995-supplementary.pdf]

Supporting Information

Ternary-Emission Molecularly Imprinted Ratiometric Fluorescence Sensor and Kit for the Rapid and Visual Detection of Enrofloxacin

Siwu Liu [1,2], Jingyi Yan [1,2], Dani Sun [2], Siyuan Peng [2], Jinhua Li [2,*] and Huaying Fan [1,*]

[1] School of Pharmacy, Key Laboratory of Molecular Pharmacology and Drug Evaluation (Yantai University), Ministry of Education, Collaborative Innovation Center of Advanced Drug Delivery System and Biotech Drugs in Universities of Shandong, Yantai University, Yantai 264005, China; liujiu645@126.com (S.L.); yjy1091897645@163.com (J.Y.)

[2] Coastal Zone Ecological Environment Monitoring Technology and Equipment Shandong Engineering Research Center, Shandong Key Laboratory of Coastal Environmental Processes, Laboratory of Coastal Environmental Processes and Ecological Remediation, Yantai Institute of Coastal Zone Research, Chinese Academy of Sciences, Yantai 264003, China; sun19961212@163.com (D.S.); 17658128539@163.com (S.P.)

* Correspondence: jhli@yic.ac.cn (J.L.); katiefhydong@sina.com (H.F.)

Supporting Figures

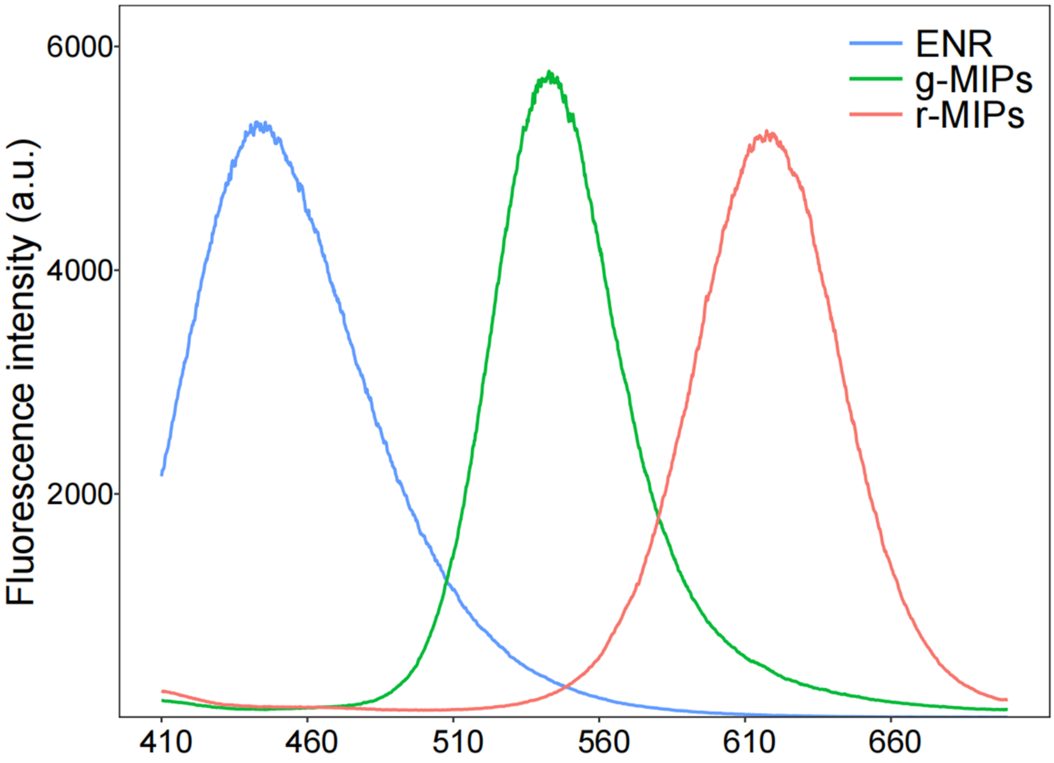

Figure S1. Fluorescence spectra of ENR, g-MIPs, and r-MIPs excited by 365 nm.

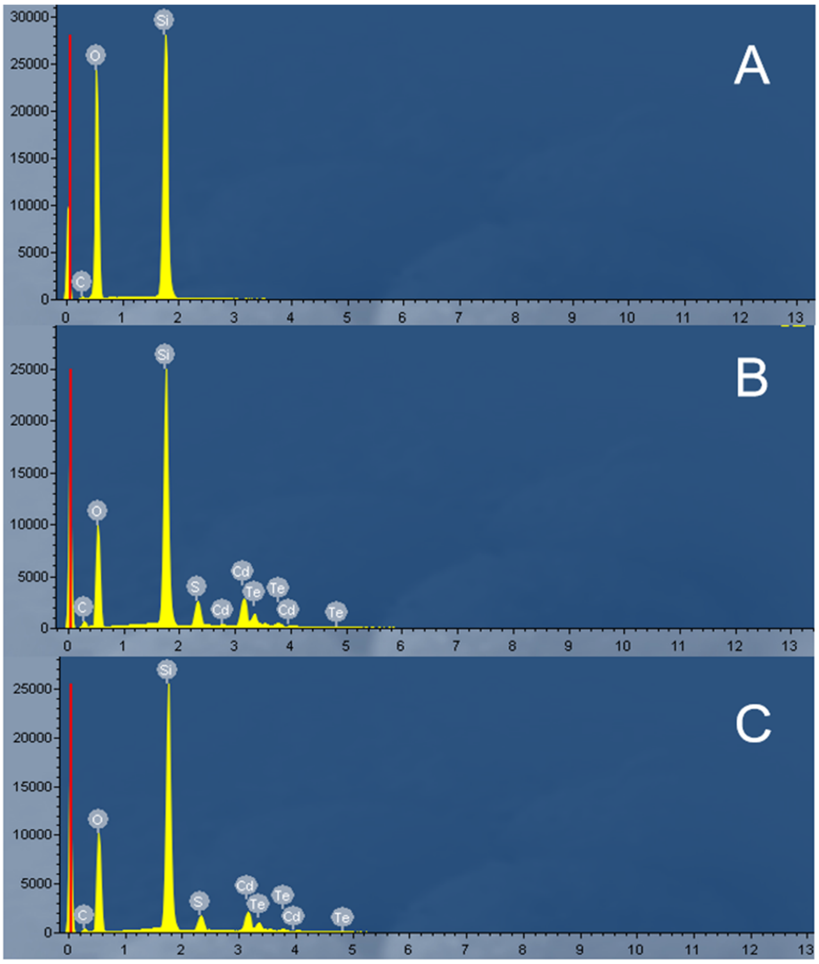

Figure S2. Energy-dispersive X-ray spectroscopy analysis: (A) SiO_2; (B) g-MIPs; (C) r-MIPs.

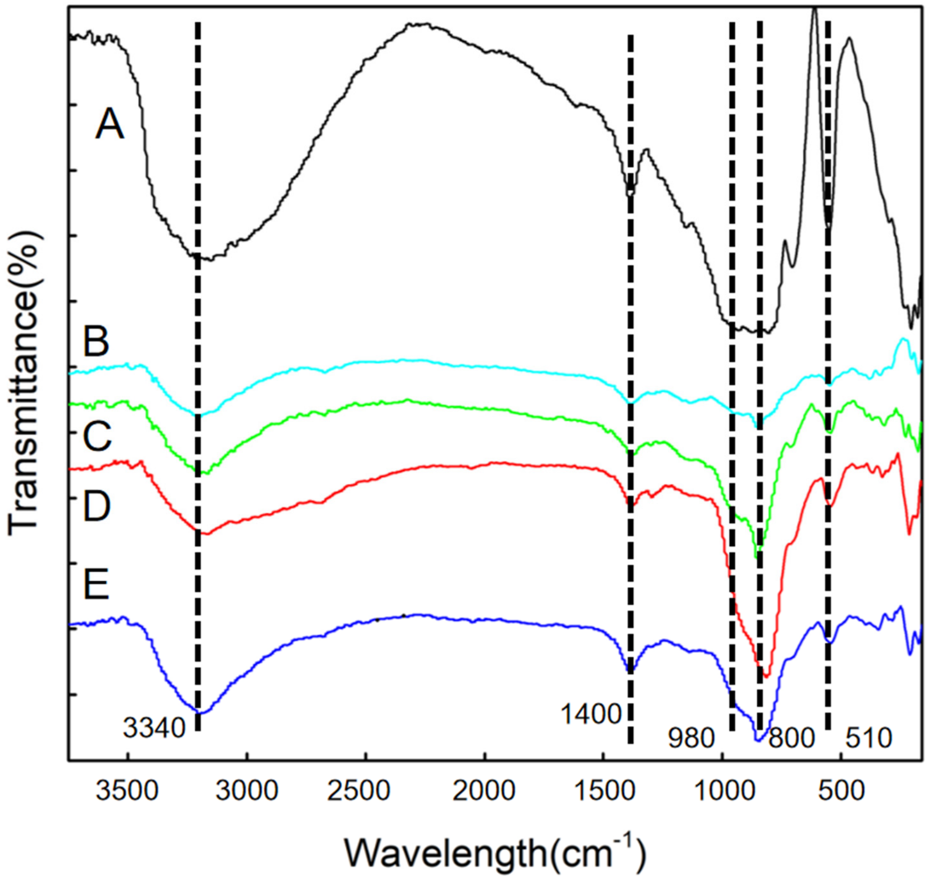

Figure S3. Infrared spectroscopic analysis: (A) SiO_2; (B) g-MIPs; (C) r-MIPs; (D) g-NIPs and (E) r-NIPs.

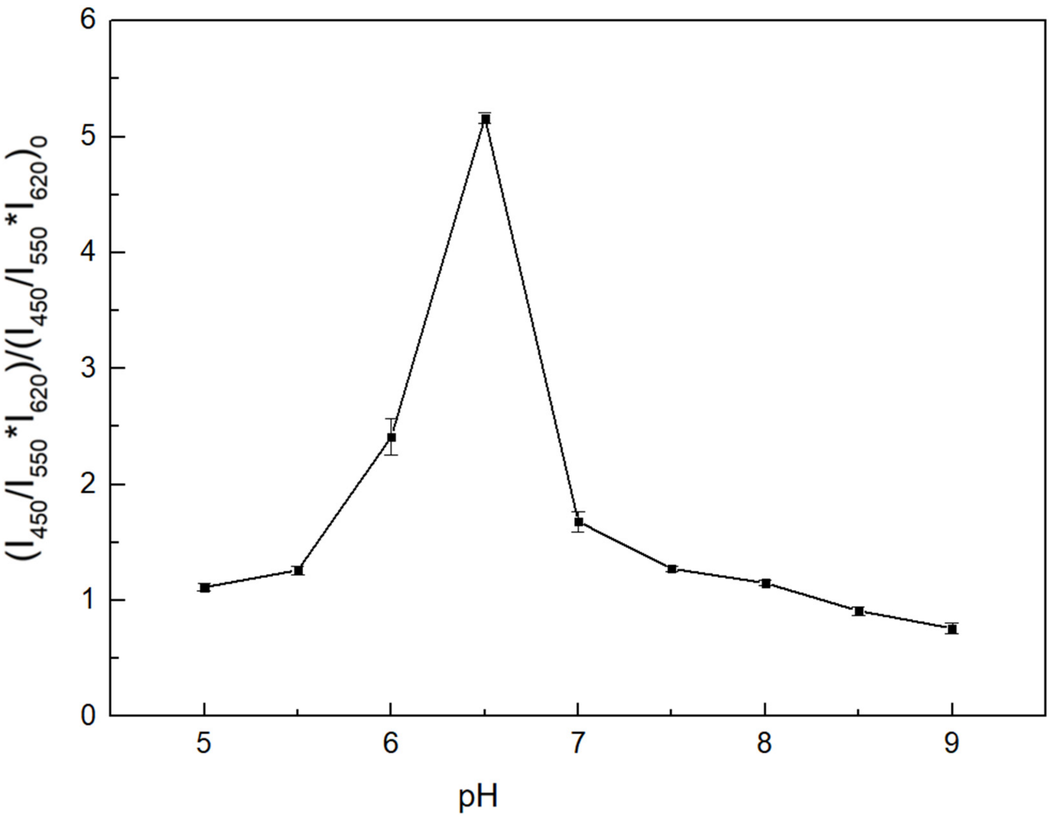

Figure S4. Effects of pH on ratio fluorescence of MIPs with 1ppm ENR.

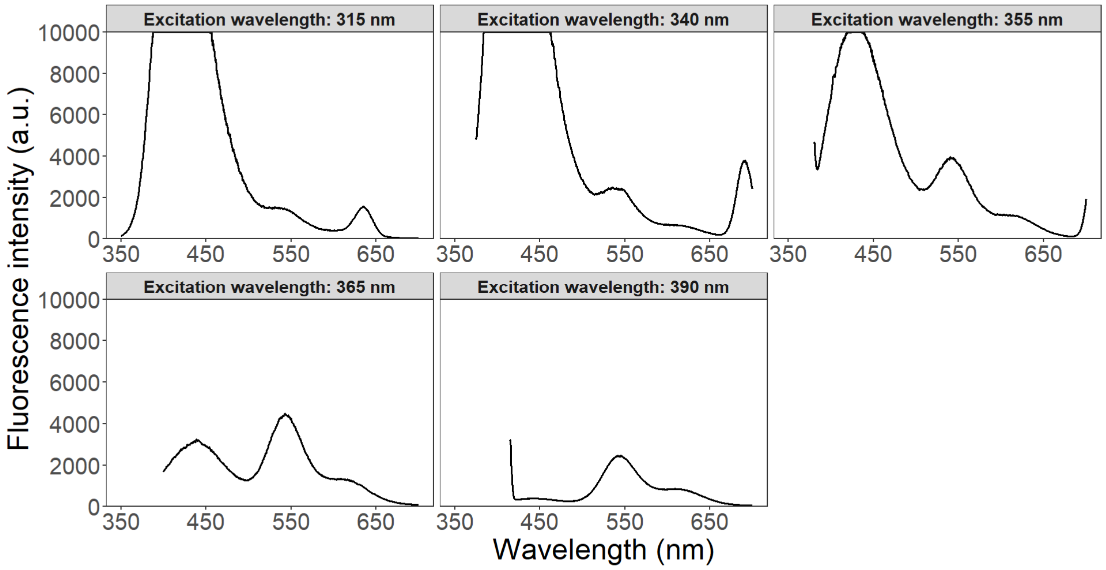

Figure S5. Fluorescence spectra of MIPs with 1ppm ENR at different excitation wavelengths.

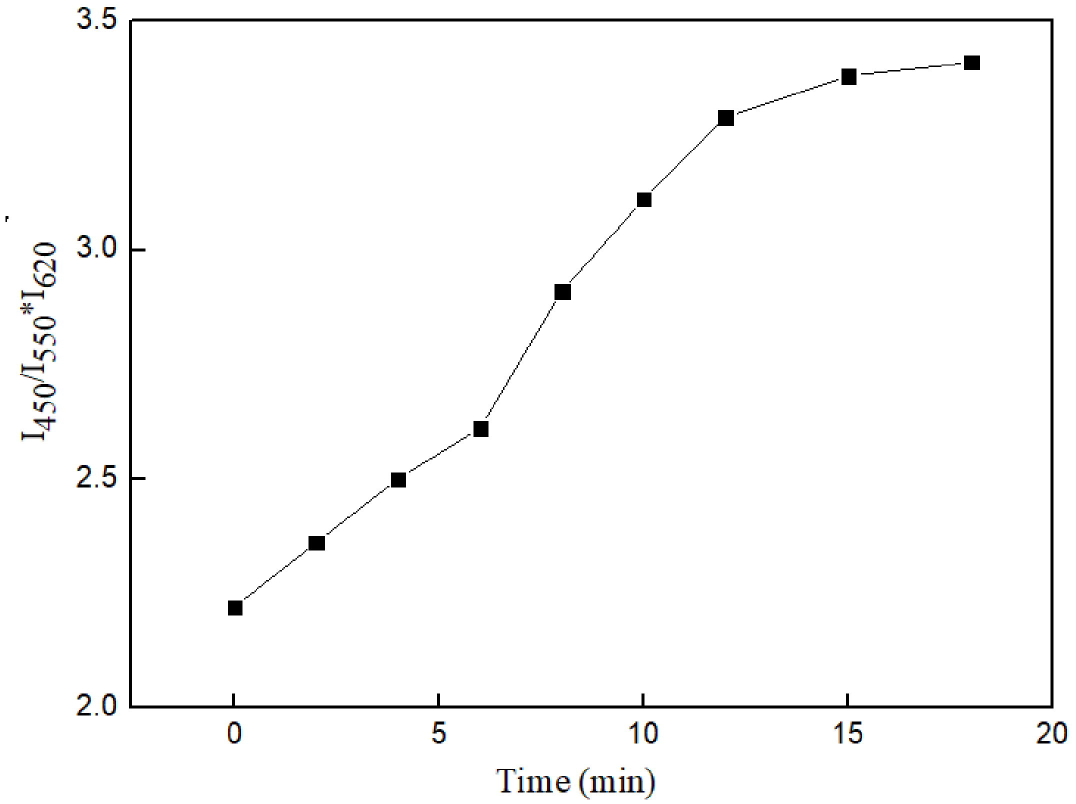

Figure S6. Responses of the MI-RFL sensor with time after adding 4 ppm ENR.

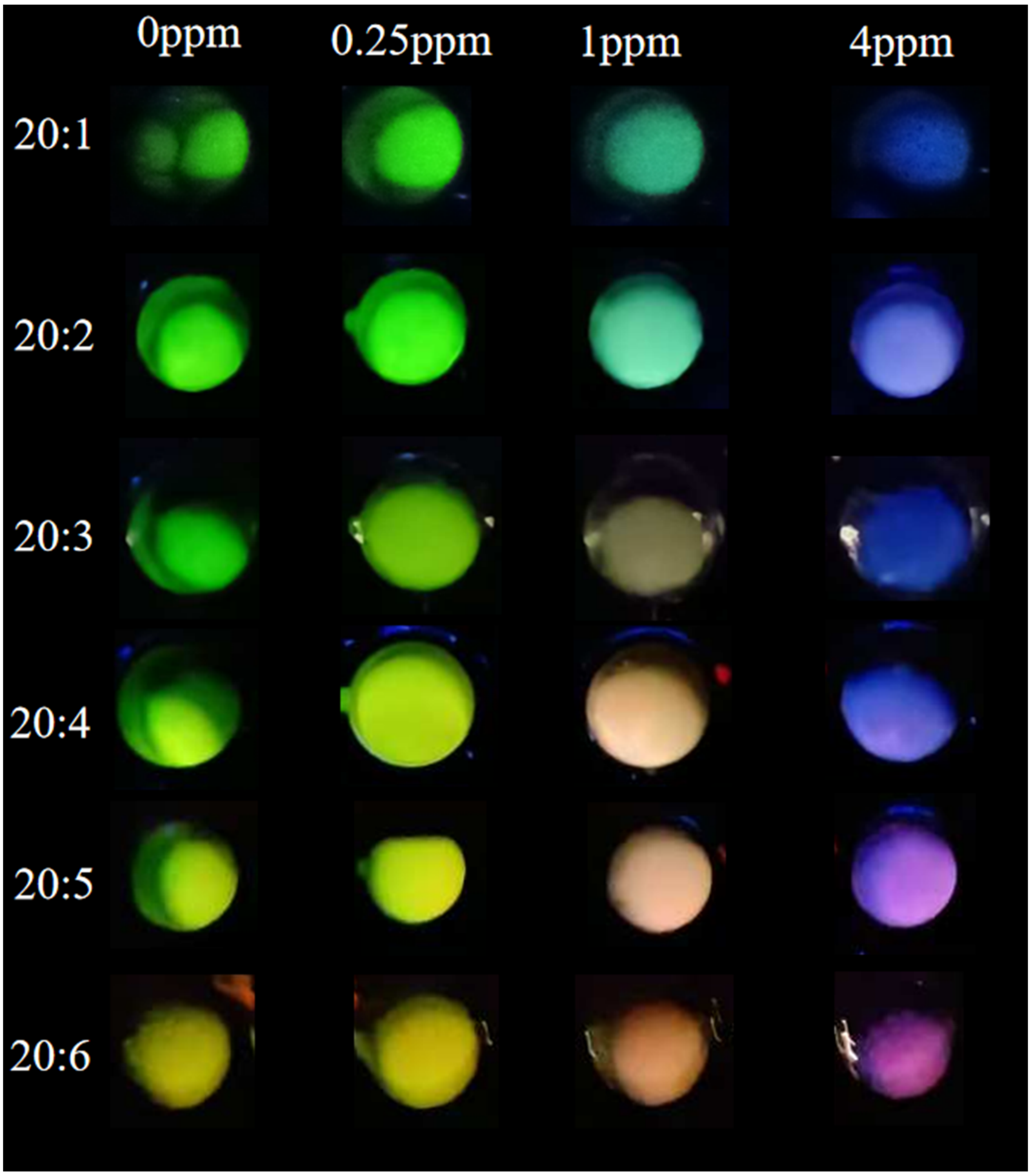

Figure S7. Effects of mixing volume ratio of green and red QDs on the fluorescence color of MIPs.

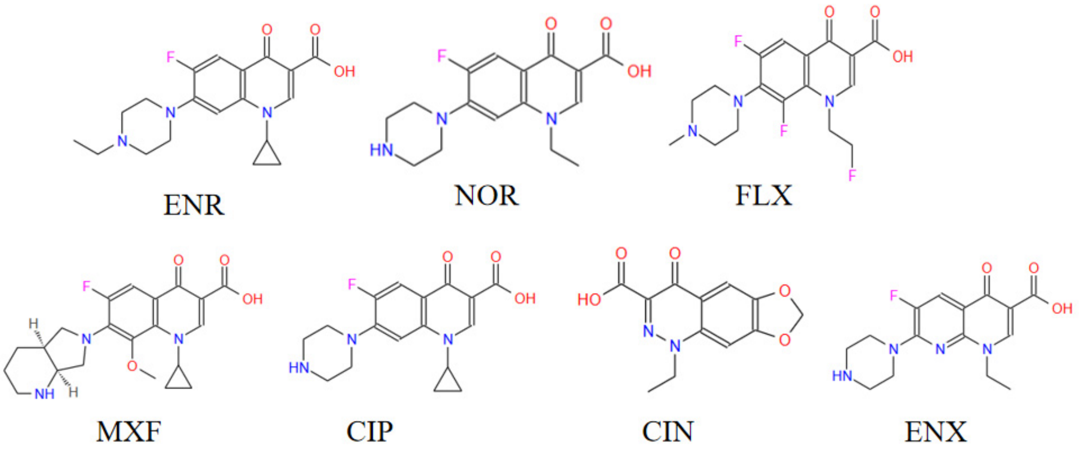

Figure S8. The structural formula of ENR and its analogs.

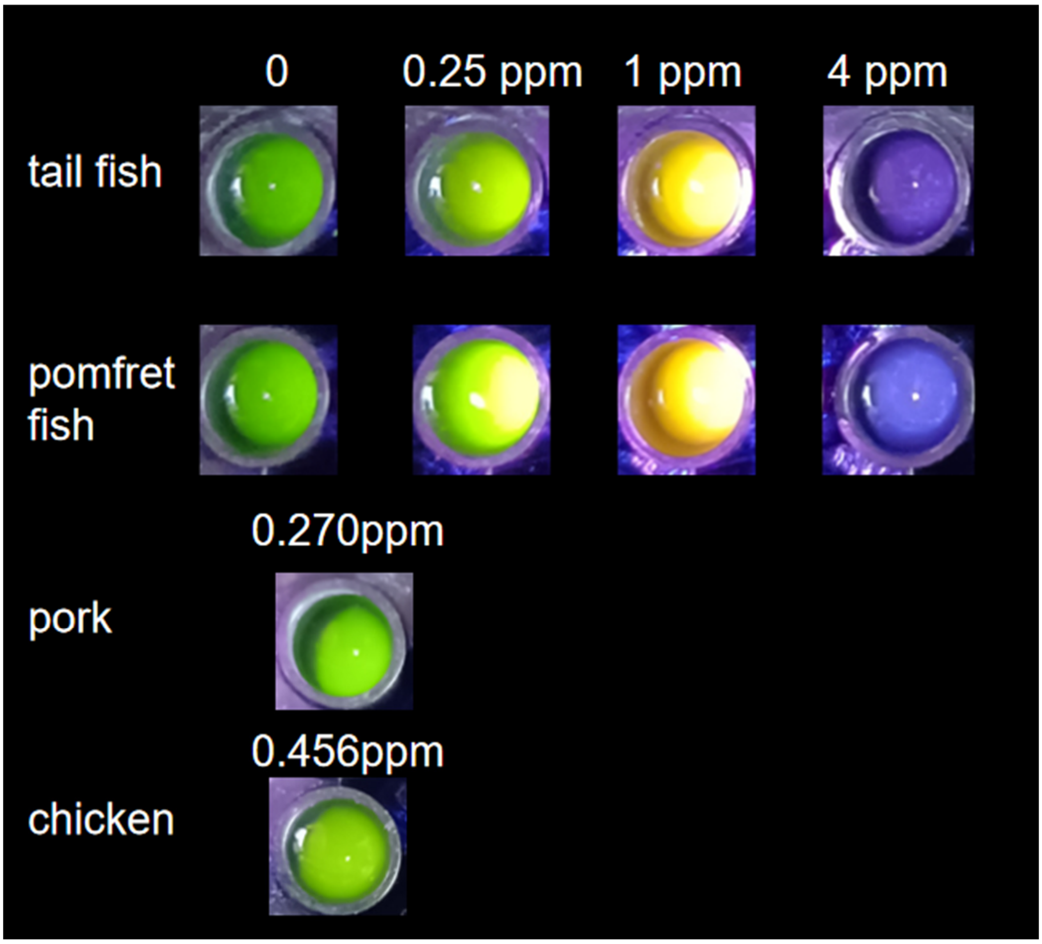

Figure S9. Fluorescence color results of different samples containing different concentration ENR.

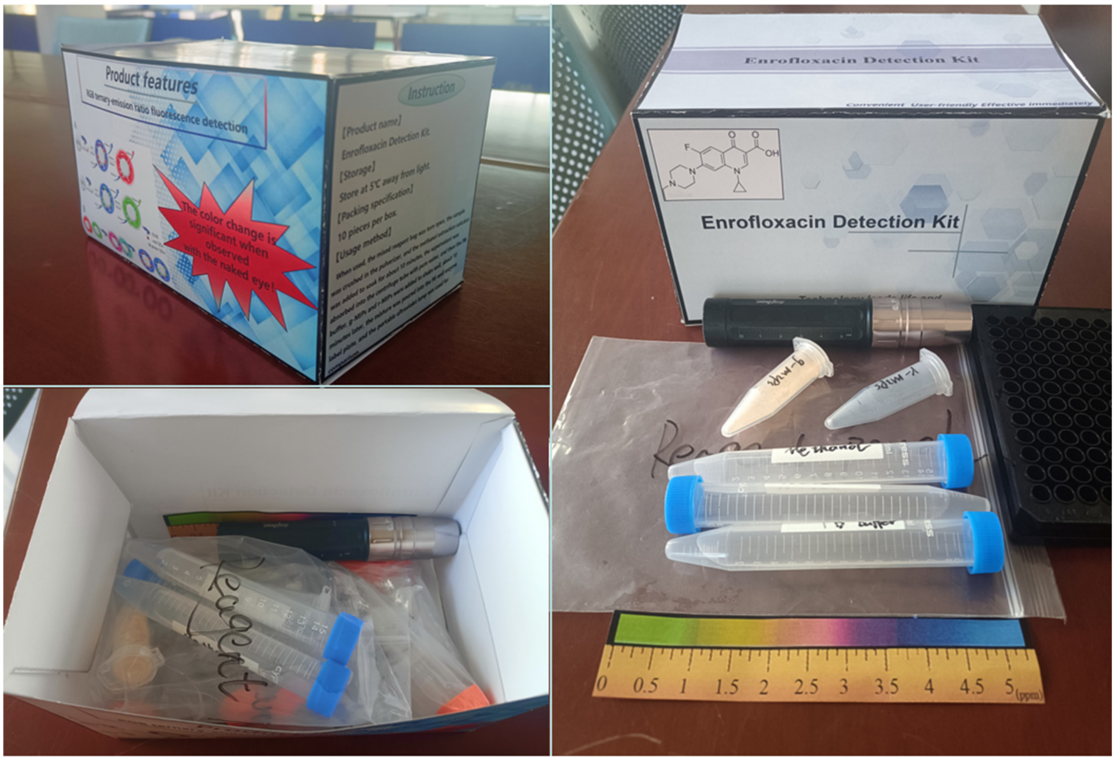

Figure S10. Photos of kit packaging and contents.

Supporting Tables

Table S1. Mobile phase elution method.

Time(min)	φAcetonitrile/%	φ(0.02% Methanoic acid)/%
0	10	90
5	10	90
15	15	85
25	20	80
40	20	80

Table S2. Intra-day and inter-day precisions of MI-RFL sensor

Intra-day precision (n=5)		Inter-day precision (n=5)	
Concentration (ppm)	RSD (%)[a)]	Concentration (ppm)	RSD (%)[b)]
0.25	2.21	0.25	4.14
1	1.49	1	6.91
4	1.77	4	7.87

a) The RSD was calculated from the ratio values of the fluorescence intensity (i.e., $I_{450}/I_{550}*I_{620}$) based on a total five consecutive measurement for each of the three concentrations in one day.

b) The RSD was calculated from the ratio values of the fluorescence intensity (i.e., $I_{450}/I_{550}*I_{620}$) based on a total five consecutive measurement for each of the three concentrations in three consecutive days.

Table S3. Comparison of the analytical performance of different MIPs based methods for ENR detection.

Detection technology	Linear range	LOD	Recovery (%)	RSD (%)	Real sample	On-site detection	Visualization	Ref.
Fluorescence	0.5-10 ng/mL (0.5–10 ppb)	0.12 ng/mL (0.12 ppb)	87.05–96.24	1.19-4.83	Perch, catfish, spanish mackerel,	×	√（blue）	28
UPLC-MS	1–1000 ng/mL (1–1000 ppb)	0.23 ng/mL (0.23 ppb)	83.79-100.68	4.46-7.35	lake water	×	×	29
Electrochemistry	10^{-5}-10^{-2} M (3.6–3600 ppm)	5x10^{-6}M (1.8 ppm)	–	–	Animals' urine	×	×	30
HPLC-UV	0.5-16 ng/mL (0.5–16 ppb)	0.24 μM (0.24 ppm)	68.88-100.29	1.6-4.4	Fish	×	×	31
Fluorescence (ternary-emission)	0.25–4 ppm	0.134 ppm	94.3-126.4	≤3.97	Seawater, bream fish, tail fish, pomfret fish, pork, chicken	√	√(green-yellow-red-purple-blue)	This work
